# Failures

**Published:** 1889-12

**Authors:** C. R. Butler

**Affiliations:** Cleveland.


ARTICLE IV.

### FAILURES.

BY DR. C. R. BUTLER, CLEVELAND.

[Read before the Ohio State Dental Society, at Cleveland, October, 1889.]

We all have them. Some may claim the contrary, and yet I make or venture the assertion, because no man has the royal heritage of infallibility. The most brillant scholar, orator, writer, artist, mechanic, and expert engineer, often fail to exhibit their best talents.

But many things are set down as failures that are not so, for two reasons: 1st, all was done that could have been under the circumstances, as it were, but with all that they come short of what they would like to accomplish. Who shall say honestly, in what have they failed?

And then the world is full of people that nominate many things as failures, because they do not accord with their notions; and those in the practiee of dentistry are not exempt from the general order. Then in the various branches of the profession, men promise too much. Perfect success in all cases, satisfaction given or no pay. There! that tooth is filled and it will last your life time; so it may if the patient don't live too long. These are many of the causes why fillings and other work fail that was not stated by a recent writer.

Another cause of failure is that some men have a notion that they can do it all, that is, be operator and patient, or so nearly so that some people act as though they have nothing to do but resist and fight the operator, instead of helping to have good operations made while in the chair. No matter how skillful the operator, he will fail to get the best results with a fractious patient; there is too much of the do at, instead of doing. If more time and care were spent in competing with ourselves, instead of thinking and talking about some one across the street as a competitor; there would be less failures in what operations we did make; much valuable time to the dentist and patients is spent often in trying to convince the latter that the operations will be made in good style for much less money than some other good operator would do it, (that has never seen the case), and only results in two failures instead of one if you failed to get the case on the list of appointments.

This may strike some as being ill-timed, or hints fit only for the novice; if so it is only an evidence that the undecorated statement of facts does not suit the notion of those that like an array of fine words, that go but very little way in putting saving fillings in teeth or curing the sick. I take it that we are here for something more than fine oratory.

And then if all were gifted or should present things in the most approved style of rhetoric, there would be no use or work for the publication committee in the way of editing the papers that are prepared by other men. Some persons are born critics, and it would be calamitous not to give them something to work upon. This is a busy world, and all dentists are not well up in dental histology, chemistry and bacteriology, and patients demand something more than to be taught these things. And if we should attempt to teach them in detail, life is too short for us to accomplish but little, so we are obliged to state things in the briefest possible manner what we can do as operator, and what they must and must not do to save their teeth. Never operate under dictation, nor commence to make a lot of fillings before removing roots or teeth that have become an offence to the other teeth and deterimental to general health, unless you want to invite failure. In many cases the best possible thing to do, is to put on the rubber-dam, clear out the cavities fairly, using an active antiseptic, then fill with oxyphosphate to remain for days, weeks, or months before attempting more permanent work.

And right here I want to say that the various cements are almost invaluable, and yet many failures are made with a material that seems so simple to handle.

It is a recognized fact among skillful and painstaking surgeons, that the nice coaptation of the wounded or incised integument, will insure the quicker union and with a minor scar. If exact technee is considered a prime factor for success in making operations upon the soft parts, how much more should it be observed in dental operations, especially in filling of teeth ?

The man that is attempting to fill teeth by gas-light, is making more failures than can be excused. Some individuals are striving to cover the whole field in dental art and science. They want to be esteemed as a first-class operator in filling, setter of crowns, bridge-worker, artificial vela, with the various modes of mounting artificial dentures, treatment of irregular jaws and teeth thrown in for a past-time.

Now it is expecting too much of the most skillfully constructed machine known to modern mechanics to have it produce No. 1 tacks and pins with the same dies. The great struggle seems to be to transmute thought through gold into gold, that we may be measured in commercial values, and so doing. We have failed thus far to make that impress upon the community at large, so that we are accepted as professional men the same as the physician. They speak of Dr. A. Oh, he is only a dentist; and however much we may do or think to the contrary, this little qualification will come in for some time to come. The inventive and business genius of the American dentist is acknowledged the world over, but this alone will not suffice to give us full fellowship in a learned profession.

Some one has said that it is much easier to talk about successes in practice than of our failures; but the left hand sand throwing by the fellow that takes delight in hauling a skeleton out of other people's closet, does not always prevent one of more hideous mean stalking to view from his private chamber.

So let us be more careful in drawing individious comparisons so long as *all* are using such incompatible material as metals to repair tooth lesion.—*Ohio Journal of Dental Science.*

